# Molecular targets in acromegaly

**DOI:** 10.3389/fendo.2022.1068061

**Published:** 2022-12-05

**Authors:** Artak Labadzhyan, Shlomo Melmed

**Affiliations:** Department of Medicine, Pituitary Center, Cedars-Sinai Medical Center, Los Angeles, CA, United States

**Keywords:** somatostatin receptor, epidermal growth factor receptor, tyrosine kinase inhibitor, cyclin-dependent kinase, granulation

## Abstract

Molecular therapeutic targets in growth hormone (GH)-secreting adenomas range from well-characterized surface receptors that recognize approved drugs, to surface and intracellular markers that are potential candidates for new drug development. Currently available medical therapies for patients with acromegaly bind to somatostatin receptors, GH receptor, or dopamine receptors, and lead to attainment of disease control in most patients. The degree of control is variable: however, correlates with both disease aggressiveness and tumor factors that predict treatment response including somatostatin receptor subtype expression, granulation pattern, kinases and their receptors, and other markers of proliferation. A better understanding of the mechanisms underlying these molecular markers and their relationship to outcomes holds promise for expanding treatment options as well as a more personalized approach to treating patients with acromegaly.

## Introduction

Acromegaly is a disease of excess growth hormone (GH) and insulin-like growth factor (IGF)-1 that is most commonly caused by a pituitary somatotroph adenoma and is associated with increased mortality (1). The pathophysiology of acromegaly, like that of other pituitary disorders, is an evolving area of research, and particularly with regard to how it informs identification of molecular therapeutic targets (2). The underlying pathogenesis likely involves disruption of cell-cycle control, alteration of signaling pathways, genetic/epigenetic changes, and abnormal hormone production. Understanding mechanisms of currently known and exploited targets, as well as other theoretical targets, allows for an intriguing opportunity for a precision approach in treating acromegaly (3).

Current pharmacological options for patients with acromegaly include somatostatin receptor ligands (SRL), growth hormone receptor agonist, and dopamine agonists (DA). These agents are mostly well tolerated, but have adverse events such as injection site reactions or gastrointestinal distress, as well as psychological/social stressors that contribute to the treatment burden (4).

Precision medicine aims to improve patient outcomes through targeted treatment employing genetic, biomarker, phenotypic, or psychosocial characteristics unique to each patient or the disease process (5). Despite challenges inherent in precision medicine, namely complexity of disease classification and expanding library of biomarker, molecular profiling of acromegaly has the potential for determining optimal drug efficacy, predicting treatment response and prognosis, and management strategies that will achieve optimal outcomes.

Here, we describe immunohistochemical, cell surface, and intracellular factors that together may comprise a subcellular basis for determining a precision medicine approach to acromegaly management (**Table 1**).

**Table 1:** Molecular targets in acromegaly.

Target	Localization	Function	Ligand	Clinical Significance	Targeted Drugs
SST2	Surface receptor	Suppress GH	SST/SRL	Marker of disease aggressiveness and tumor response	Octreotide and lanreotide; pasireotide to a lesser degree
SST5	Surface receptor	Suppress GH	SST/SRL	Marker of disease aggressiveness and tumor response	Pasireotide; octreotide and lanreotide to a lesser degree
D2R	Surface receptor	Lower GH	DA	Limited; marker of DA response	Cabergoline and bromocriptine
GHR	Surface receptor	Induction of IGF-1	GH	Limited	Pegvisomant, Cimdelirsen (investigational)
E-cadherin	Surface protein	Cell adhesion		Marker of disease aggressiveness and tumor response	TBD
β-arrestin	Surface protein	Receptor modulator	GPCR	Limited; marker of tumor response	TBD
EGFR	Surface TK receptor	Cell proliferation	EGF, TFG-α	Limited	TKIs such as gefitinib, lapatinib
Granulation	Secretory granules			Marker of disease aggressiveness and tumor response	TBD
AIP	Intracellular	Cell-cycle regulator		Marker of disease aggressiveness and tumor response	TBD
GNAS	Intracellular	Cell-cycle regulator		Limited	TBD
PTTG	Intracellular	Cell-cycle regulator		Limited	TBD
ZAC1	Intracellular	Transcription factor Cell proliferation		Marker of disease aggressiveness and tumor response	TBD
Ki-67	Nuclear antigen	Proliferation marker		Marker of disease aggressiveness and tumor response	TBD
p21	Intracellular	CDK inhibitor Cell senescence		Marker of disease aggressiveness and tumor response	TBD
p27	Intracellular			Limited	TBD
p16	Intracellular	CDK inhibitor		Limited	TBD
p53	Intracellular	Tumor suppression Cell senescence		Limited	TBD

TBD, to be determined.

## Pathophysiology

Differentiation of GH-producing somatotroph cells in the anterior pituitary is determined by the PIT1 (POU1F1) transcription factor. Somatotroph adenomas are almost invariably sporadic, but familial tumors occur in very rare disorders such as MEN1, Carney complex, or X-LAG acrogigantism (6). A notable cause of familial isolated pituitary adenomas is germ-line mutation in the *AIP* gene (7).

Tumorigenesis involves dysregulation of cell proliferation and GH production through transcriptional, hormonal, and other growth stimulating factors. GH gene transcription and secretion is mediated by intracellular cAMP, and alterations in cAMP signaling leads to dysregulated GH production (4). Activating *GNAS* (guanine nucleotide binding protein, alpha stimulating activity polypeptide 1 gene) mutations, present in up to 40% of sporadic tumors, leads to constitutive cAMP activation and consequent excess GH production (8). However, classic oncogene mutations are not encountered in the pathogenesis of acromegaly.

**Figure 1:**
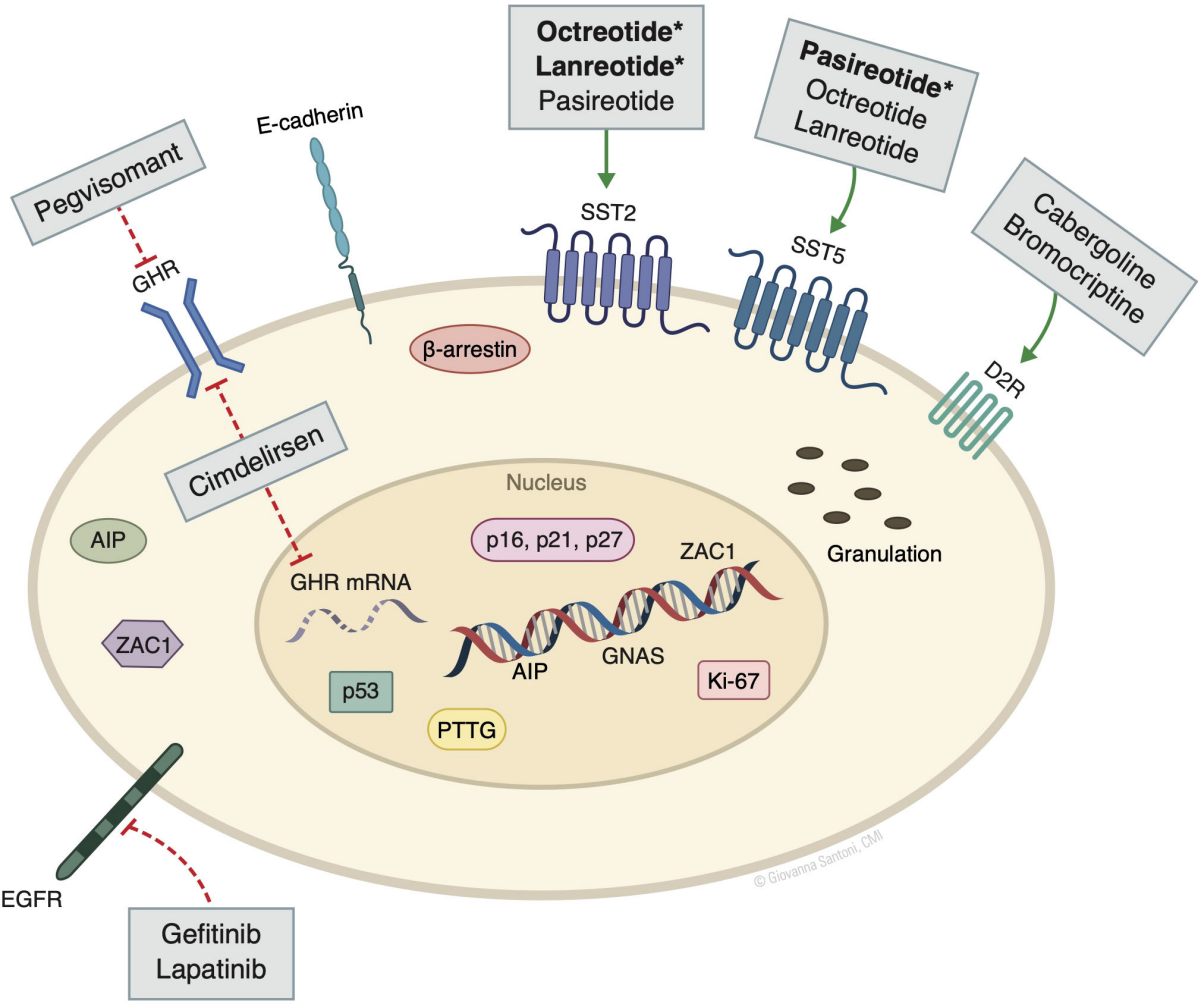
Molecular markers and targeted drugs. AIP, aryl hydrocarbon receptor-interacting protein; D2R, dopamine receptor subtype 2; GHR, growth hormone receptor; GNAS, guanine nucleotide binding protein, alpha stimulating activity polypeptide 1 gene. SST, somatostatin receptor; PTTG, pituitary tumor–transforming protein; ZAC1, zinc finger regulator of apoptosis and cell cycle arrest. Bolded^*^ denotes the higher/predominant affinity of drug to receptor. The action of pegvisomant on GHR is mainly peripheral and not in the pituitary. ^©^ Giovanna Santoni, CMI. Used by permission.

Several other non-oncogenic factors play important pathogenic roles, including stimulatory signals from central and peripheral hormones, and disruption of proteins and kinases that regulate the cell cycle. Peripheral sex steroids and hypothalamic growth hormone releasing hormone (GHRH), if dysregulated, may induce GH production through constitutive cAMP production, as well as promote tumor cell proliferation. Disruption of cell cycle regulatory factors such as cyclin-dependent kinases (CDK), CDK inhibitors (e.g., p21, p27), and pituitary tumor–transforming protein (PTTG) also allow for pituitary tumor growth (4) (**Figure 1**).

Cell surface receptors for somatostatin, GHRH, and dopamine play a less direct role in pathogenesis, but are important mediators of stimulatory and inhibitory signals, and their balance is required to maintain normal GH secretory physiology.

## Cell surface receptors as molecular targets

### Somatostatin receptors

The hypothalamic-pituitary axis involves the interplay of tightly regulated hormone action through positive and negative signaling. Somatotroph GH production is attenuated by hypothalamic somatostatin, which suppresses timing and amplitude of GH secretory pulses by binding to somatostatin receptors (SST) (4), a family of G protein-coupled transmembrane receptors (GPCR) with five known subtypes (SST1-5), each with differing binding and hormone modulating capacity. SST2 and SST5 are most abundantly expressed receptors on somatotrophs and are the primary targets of adenoma-directed medical therapy (9, 10).

SST2 is expressed in brain, pituitary, stomach, kidney, and intestines (11). It signals to suppress mainly GH, and less compellingly thyroid-stimulating hormone (TSH), adrenocorticotrophin, and prolactin (12). The prevalent expression of SST2 in GH-secreting adenomas (9) makes it a prime target for treating acromegaly. The SRLs octreotide and lanreotide preferentially bind to SST2, thereby suppressing GH expression (1, 13). Disease control with these agents, however, can be variable with a reported mean response rate of approximately 55% (14).

SST5 is primarily expressed in the pituitary but is found as well in the spleen, intestines, and pancreas (15–17). SST5 regulates GH and TSH (12). Unlike with octreotide and lanreotide, the SRL pasireotide predominantly targets and binds with high affinity to SST5. It has shown to be more efficacious in patients resistant to other SRLs (18, 19), but it is associated with an increased risk of hyperglycemia, likely due to the role of SST5 in regulating glucose homeostasis through paracrine regulation of intestinal GLP-1 (20). Intuitively, lowering circulating GH also enhances insulin sensitivity thereby countering some of these hyperglycemic effects, but the risk remains and potentially limits its use.

Immunohistochemical expression of SST2 and SST5 may predict treatment outcomes, especially as expression is less abundant in more aggressive adenomas that are less prone to treatment responsiveness (21). For example, we showed that SRL treatment efficacy is typically high in patients with low or intermediate tumor aggressiveness, and these patients show concordant abundant SST2 expression. By contrast, SST2 expression is lower in patients with more aggressive and highly unresponsive tumors (21). Others have reported that response rate to SRL may be as high as 81% in the presence of SST2 expression compared with no response observed in the absence of SST2 expression (22, 23). Further, a higher SST5:SST2 ratio correlates with better response to pasireotide and resistance to octreotide/lanreotide (24–26). Of note, epigenetic factors, such as the natural antisense transcript SST5-AS, has been shown to alter SST5 expression, potentially influencing adenoma behavior and treatment response (27).

Although not routinely evaluated by clinical pathologists, SST expression may be a valuable molecular marker for precision-based acromegaly treatment, as decision for repeat surgery, radiotherapy, or choice of a specific SRL could be better guided with knowledge of SST2 and SST5 expression.

### Growth hormone receptor

Human growth hormone receptor (GHR) is a transmembrane protein structurally related to a family of cytokine receptors (28, 29). Binding of GH leads to GHR dimerization and activation through the JAK/STAT pathway, leading to induction of IGF-1 (30). GHR is the target of the peripheral GHR antagonist pegvisomant, which is highly effective in treating acromegaly both as monotherapy and in combination with SRL (31, 32). A novel drug in development, Cimdelirsen (IONIS-GHR-LRx; ISIS 766720), which is an antisense molecule that acts by reducing GHR synthesis in the liver, has also shown promise in treating acromegaly (33). Polymorphisms in GHR, such as d3-GHR, has been studied in acromegaly but correlation with clinical features or therapeutic outcomes has not been consistent (34).

GHR expression is observed in normal somatotroph cells and to a lesser degree in somatotroph adenomas (35, 36). Pegvisomant may in part affect growth hormone production through direct action on GHR on somatotroph cells without impacting cell proliferation (37). However, the value of GHR as either a peripheral or central molecular marker for predicting treatment response is uncertain.

### Dopamine receptors

Dopamine receptors (DR) comprise five GPCR subtypes numbered D1R through D5R and are present abundantly in the central nervous system, and peripherally in the pituitary, kidney, and vasculature (38, 39). D2R is present in two isoforms of equal activity and distribution, and is the predominant DR found in normal pituitary as well as in somatotroph and lactotroph adenomas (25).

The downstream cellular action of activated DR includes inhibition of adenylyl cyclase and activation of potassium channels, leading to inhibitory action of hypothalamic dopamine on prolactin production (40). In somatotroph adenomas, dopamine lowers GH production (41). This is likely the mechanism underlying the modest efficacy of DA monotherapy in the treatment of mild acromegaly (42) as expression of DR on somatotroph adenoma may have some value in predicting clinical response to SRL (25) yet serum prolactin level does not correlate with GH responses to cabergoline (42).The very limited value of using D2R as a therapeutic marker for acromegaly is limited to identifying patients with mild disease who may respond to DA as monotherapy or as an adjunct to SRL therapy.

## Other cell-surface molecules

### E-cadherin

E-cadherin is a transmembrane adhesion protein found on epithelial cells, and its loss is implicated in invasiveness and metastasis (43). Its expression has been suggested to be functional for somatotroph adenoma growth (44), but this association is modest, likely because other factors that promote cell senescence (45) prevent low expression of E-cadherin from causing transformation of pituitary adenomas to malignant tumors. Decreased E-cadherin expression in somatotroph tumors correlates with larger size, invasiveness, and response to SRL treatment (44). Sparsely granulated somatotroph adenomas, which are more likely to be aggressive (discussed below), also show lower E-cadherin expression compared to densely granulated tumors (46, 47). The association between low E-cadherin expression and poor SRL response seems independent of SST2 expression but may correlate inversely with SST5 expression (48). Such correlations between histological and cell surface markers provide an interesting intersection of molecular markers of tumor aggressiveness. However, direct relationships of these factors are not known, and they may simply represent the phenotypic components of more aggressive tumors.

### Epidermal growth factor receptor (EGFR)

The EGFR family of tyrosine kinase receptors including EGFR (ErbB1 and HER1), p185^Her2/neu^ (ErbB2 and HER2), ErbB3 (HER3) and ErbB4 (HER4), and their activating ligands including EGF and transforming growth factor-α are expressed in lacto-somatotroph cells and are potential targets for treatment (49). EGFR tyrosine kinase inhibitors (TKI) such as gefitinib, lapatinib, and canertinib have shown promising results in patients harboring aggressive corticotroph and lactotroph adenomas (50, 51). Although EGFR and EGF are abundantly expressed in somatotroph adenomas, trials evaluating TKI in treatment of aggressive acromegaly are lacking.

### β-Arrestin

β-arrestin binds to GPCR and thereby regulates signaling (52). Results of studies on the role of β-arrestin in responsiveness of somatotroph adenomas to therapy has been mixed. In a study of 31 somatotroph adenomas, lower expression of β-arrestin-1 correlated with improved responsiveness to SRL (53), and a similarly sized study showed an inverse relationship between low β-arrestin-1/2 and SST2 expression, as well as a more favorable long-term response to SRL treatment (54). However, this relationship was not observed in another study of 40 patients, which showed no correlation of β-arrestin with SST2, SST5, or D2 expression, nor an association between β-arrestin and SRL response or tumor invasiveness (55). Therefore, the significance of β-arrestin as a molecular target for acromegaly remains unclear.

## Granulation

Somatotroph adenomas are classified in two immunohistochemical subtypes - sparsely or densely granulated – with important clinical implications (56). The granulation pattern refers to the density of intracellular GH secretory granules as seen on electron microscopy, and is distinguished immunohistochemically by expression of cytokeratin expression, which is a cytoplasmic fibrous protein belonging to the family of intermediate filament proteins comprising the cytoskeleton of the cell (57, 58). As a dense granulation pattern is seen in normal somatotroph cells, densely granulated tumors more closely resemble nontumorous cells, while sparsely granulated tumors with scattered small secretory granules resemble poorly differentiated cells (59). The clinical outcomes associated with granulation pattern is generally consistent with outcomes associated with tumors harboring normal or poorly differentiated cells. Densely granulated adenomas are smaller at diagnosis, express higher levels of SST2, and are more responsive to treatment; therefore, these are less aggressive tumors. Conversely, sparsely granulated tumors are more likely to be aggressive: they are larger at diagnosis, show sparsity of SST2 expression, and are less responsive to treatment (21, 22).

## Cell-cycle regulatory factors

### Aryl hydrocarbon receptor-interacting protein (AIP)

Inactivating mutations of the tumor suppressor gene *AIP* in pituitary tumors is associated with familial acromegaly syndromes and is rarely seen in sporadic acromegaly (7). Familial acromegaly involving *AIP* mutation is more aggressive and less responsive to SRL (60). AIP expression has been associated with dense granulation (61), and inversely correlates with Ki-67 expression (62), suggesting a pro-proliferative state.

The mechanism of AIP mutation and tumorigenesis likely involves alterations in PDE4 phosphodiesterases as well as defective cAMP signaling. Specific PDE4 isoforms, PDE4A4 and PDE4A8, are under-expressed in normal pituitary and overexpressed in somatotroph adenomas, suggesting that disruptions of PDE4-AIP interaction play a role in tumorigenesis (63). Dysfunction in cAMP signaling through defective and decreased expression of Gai subunits, which help regulate AIP mediated cAMP signaling, also plays a key role (64). The mechanism of interplay between expression of AIP and cell-surface molecular receptors requires further study and may yield novel sub-cellular targets for treatment.

### GNAS

Mutations in GNAS are present as somatic mutations in up to 40% of sporadic somatotroph adenomas and as mosaic mutations in McCune-Albright syndrome, a genetic disorder characterized by skin and bone manifestations as well as increased incidence of acromegaly (65). The impact of GNAS mutation on treatment response is uncertain. Though a meta-analysis showed a greater GH reduction in response to SRL treatment in GNAS mutations (66), a more recent study did not show a significant difference in SRL response and GNAS mutations (67). Pegvisomant is also an effective treatment option for acromegaly in patients with MAS (68); however, whether GNAS mutation influences treatment outcome between different available therapies is not known.

### PTTG

PTTG is a homolog of securin proteins that prevent sister chromatin separation (69), and regulate the cell cycle through interaction with p53 (70). Over 70% of somatotroph adenomas overexpress PTTG, and this expression is an important component of cell senescence (71). PTTG expression may correlate with aggressiveness across different pituitary adenoma types (72), but it’s role as a molecular marker for clinical use in somatotroph adenoma characterization and targeted treatment warrants further investigation.

### ZAC1

Zinc finger regulator of apoptosis and cell cycle arrest (ZAC1) is a tumor suppressor that attenuates cell proliferation (73). The antiproliferative property of SRL therapy may be mediated in part through increased *ZAC1* gene expression (74), with evidence that suggests increased AIP expression is the linking mechanism (75). The lack of difference in ZAC1 expression between densely and sparsely granulated adenomas implies that ZAC1 does not play a significant role in tumor aggressiveness (57).

### Ki-67

In the 2017 WHO Classification of Tumors of the Pituitary Gland, the term or classification of atypical adenoma, which was defined partly by Ki-67 proliferative index, was no longer recommended (76). However, Ki-67 may still hold value in predicting aggressiveness and treatment response in somatotroph adenomas.

Most non-aggressive adenomas show a Ki-67 proliferative index of <3% (21), and low Ki-67 expression has been associated with more favorable SRL response (67). Additionally, Ki-67 index correlates inversely with cavernous sinus invasion, surgical cure, and response to medical therapy (77). SRL treatment may also alter Ki-67 expression as evidenced by lower adenoma Ki-67 values observed in patients treated chronically with octreotide (78).

### Cyclin-dependent kinase inhibitors

CDK and their inhibitors may serve as markers of somatotroph adenoma subtypes (4, 79). p21, a CDK inhibitor that plays a key role in cell senescence, is activated to maintain the benign nature of somatotroph adenomas (45, 71). Increased expression of p21 is associated with less aggressive somatotroph adenomas, likely exerting a dampening effect on cell proliferation (21). However, its impact in predicting curative success of surgery may be limited. In a series of 55 patients undergoing surgical adenoma resection, p21 overexpression did not correlate with biochemical remission after surgery (80), likely because the skill and volume of the surgeon, is a primary predictive factor for surgical success, more so than any one particular molecular factor (81).

The value of other CDK inhibitors such as p16 and p27 as molecular drivers in acromegaly are less certain. p16 expression is low or undetectable in all pituitary adenoma types and does not correlate with somatotroph tumor aggressiveness (21, 82). Low or absent p27 expression seems to play an especially important role in aggressive corticotroph adenomas as well as malignant pituitary tumors. Although p27 expression is also lower in somatotroph adenomas, its correlation to tumor aggressiveness or response in acromegaly is less clear (83). Despite limitations, CDK inhibitors may serve as compelling molecular predictors of tumor proliferative growth and as targets for development of novel drugs that target CDK pathways.

### p53

p53-mediated tumor suppression in pituitary pathology acts through the p53/p21 senescence pathway (71). However, p53 is not reliable in predicting tumor aggressiveness. Somatotroph adenoma p53 overexpression has not shown significant association with tumor invasiveness or response to treatment (84–86).

## Conclusion

The molecular profile of acromegaly affords an array of potential targets that may predict tumor aggressiveness or treatment response. Somatostatin receptors are the most well studied markers, and SST2 and SST5 are the targets of standard of care SRL treatment, and varying expression levels of these receptors also reliably predict response to specific types of SRL as well as overall resistance to treatment. The cell surface receptors GHR and DA also serve as targets for the highly effective GHR antagonist pegvisomant and for DA used primarily in an adjunctive treatment setting, respectively, but there is less evidence regarding their use as markers of tumor aggressiveness. Cell-surface molecules and cell-cycle regulatory factors involved in the complex interplay of cell signaling and cell cycle regulation in somatotroph tumors, including E-cadherin, CDK inhibitors, and EGFR TKIs, hold potential as targets for new therapies in the future.

## Author contributions

AL conducted the initial literature review and prepared the first draft of the manuscript. All authors reviewed and approved the final version of the manuscript and made the decision to submit.

## Funding

Supported by NIH grants R01DK113998 and T32DK007770, and by the Doris Factor Molecular Endocrinology Laboratory at Cedars-Sinai.

## Conflict of interest

The authors declare that the research was conducted in the absence of any commercial or financial relationships that could be construed as a potential conflict of interest.

## Publisher’s note

All claims expressed in this article are solely those of the authors and do not necessarily represent those of their affiliated organizations, or those of the publisher, the editors and the reviewers. Any product that may be evaluated in this article, or claim that may be made by its manufacturer, is not guaranteed or endorsed by the publisher.
